# The Association Between Comorbidities and Prescribed Drugs in Patients With Suspected Obstructive Sleep Apnea: Inductive Rule Learning Approach

**DOI:** 10.2196/39103

**Published:** 2023-01-30

**Authors:** Daniela Ferreira-Santos, Pedro Pereira Rodrigues

**Affiliations:** 1 Department of Community Medicine, Information and Decision Sciences Faculty of Medicine University of Porto Porto Portugal; 2 Center for Health Technology and Services Research Porto Portugal

**Keywords:** association rule mining, drug, electronic health records, obstructive sleep apnea, problem list, comorbidities, prescribed drugs, sleep apnea, disease-drug associations, diagnoses, clinical data, EHR

## Introduction

Current evidence suggests that patients with accurate and complete electronic health records (EHRs) may receive higher quality care than patients with gaps in their EHRs, which can be critical for quality measurement and research [1].

This paper explores the automated acquisition of disease-drug associations in documents written daily by health care professionals, using the available drug information to help fill missed diagnoses in patients with suspected obstructive sleep apnea (OSA).

## Methods

We manually extracted 26 predictive variables plus prescribed drugs (World Health Organization Anatomical Therapeutic Chemical [ATC] classification system [2]) and the apnea-hypopnea index, within a consecutive cohort of 619 patients referred to polysomnography at the University Hospital Center of São João, Portugal between 2011 and 2019 who were older than 18 years and had suspected OSA.

Disease-drug rules were induced [3,4], and we selected those with a lift higher than 1 [5] and a confidence higher than 85% [1] if the rule was present in both the “drug-to-disease” and “disease-to-drug” association sets. Rules were evaluated by a clinical expert to interpret the clinical significance of each disease-drug pair.

## Results

**Figure 1:**
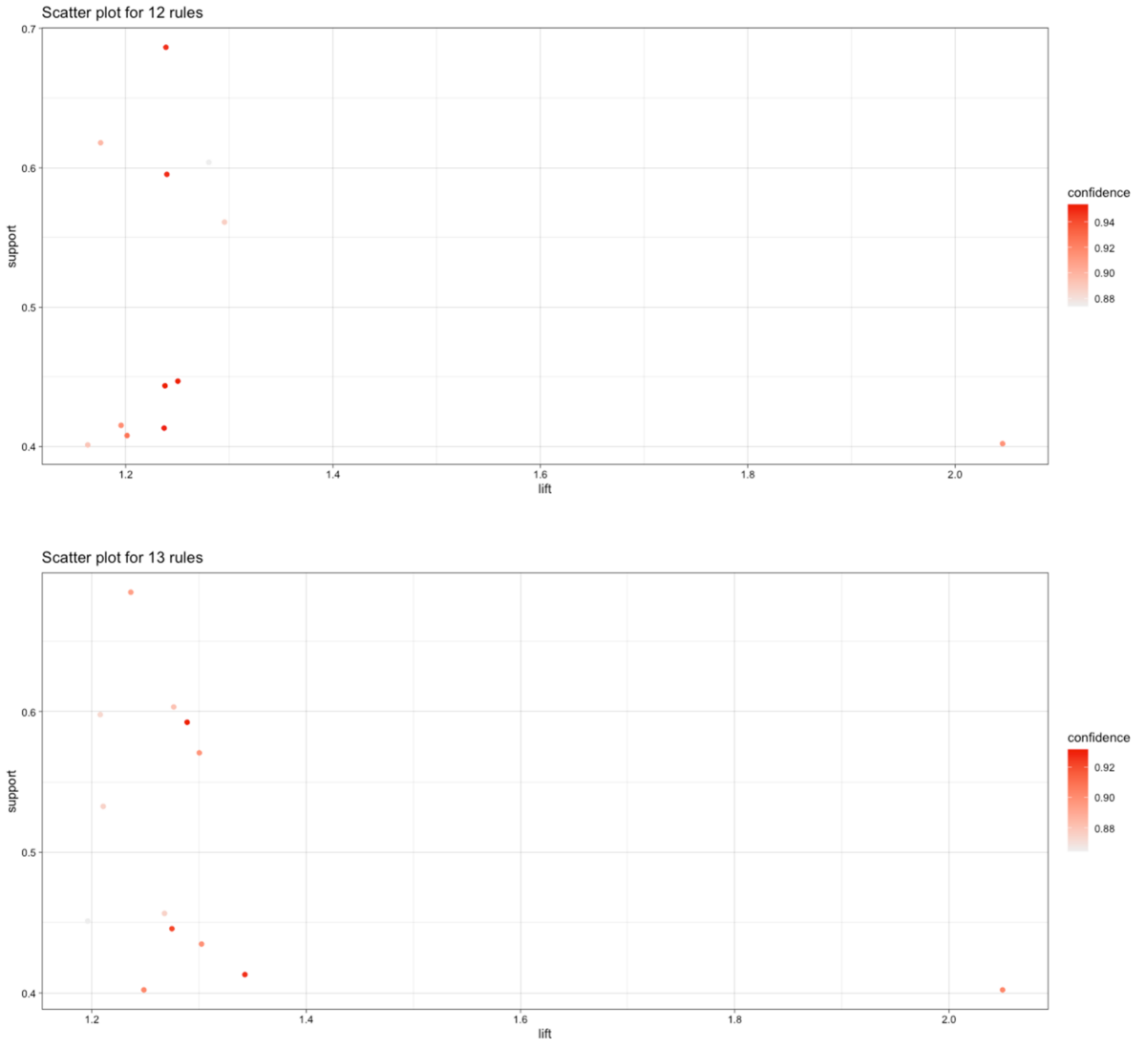
Association rules for prescribed drugs to diseases (upper image) and for diseases to prescribed drugs (lower image).

A total of 481 patients, with 34 described diseases and 50 prescribed drugs, had a variation in missing data of 0% to 97%. We found a total of 29 disease-drug associations with a lift higher than 1 (15 rules for “drug-to-disease” and 14 for the “disease-to-drug”), but only 25 had a confidence higher than 85%. The obtained rules are listed in Table 1 (and Figure 1) by decreasing order of lift, alongside support and confidence.

The rule found with the highest support (68%) associates C09 and arterial hypertension, which also presented the highest confidence in all rule sets, 95% (ie, of the patients that took C09, 95% had arterial hypertension). The highest lift value was 2.05 for the rule associating A10 and diabetes (ie, knowing that a patient took A10, the probability of having diabetes is 2.05 times higher than that of having diabetes not knowing if they are taking A10).

Three strong disease-drug rules were found, namely, the association between A10 → diabetes, C10 → dyslipidemia, and C09 → arterial hypertension. The initial proportion of missing data for diabetes was 47%, for dyslipidemia was 35%, and for arterial hypertension was 23%. These proportions reduced to 46%, 31%, and 21%, respectively, after disease-drug rules were applied to our data set. In our data set, 90% (n=74) of patients with diabetes reported A10 drug prescriptions, 88% (n=111) of patients with dyslipidemia reported C10 drug prescriptions, and 89% (n=126) of patients with arterial hypertension reported C09 drug prescriptions.

Additionally, the A10, C10, and C09 second-level ATC codes also appeared related to other diseases (eg, A10 → arterial hypertension). Nevertheless, these rules are not presented in both the “drug-to-disease” and “disease-to-drug” sets.

**Table 1:** Association rules for prescribed drugs to diseases and diseases to prescribed drugs (italics represent a strong rules association).

		Lift	Support	Confidence
**Drug → disease**				
	*A10*^a^ → *diabetes*	*2.05*	*0.40*	*0.91*
	C09^b^ and C10^c^ → dyslipidemia	1.30	0.56	0.89
	*C10* → d*yslipidemia*	*1.28*	*0.60*	*0.87*
	*C09* → a*rterial hypertension*	*1.24*	*0.68*	*0.95*
	C09 and A02^d^ → arterial hypertension	1.24	0.45	0.95
	C09 and N06^e^ → arterial hypertension	1.24	0.45	0.95
	C09 and C10 → arterial hypertension	1.24	0.60	0.95
	C09, C10, and A02 → arterial hypertension	1.24	0.41	0.95
	A10 → arterial hypertension	1.21	0.41	0.93
	C10 and A02 → arterial hypertension	1.19	0.41	0.92
	C10 → arterial hypertension	1.17	0.62	0.90
	N06 and C10 → arterial hypertension	1.16	0.40	0.89
**Disease → drug**				
	*Diabetes* → *A10*	*2.05*	*0.40*	*0.90*
	Diabetes → C10	1.34	0.41	0.93
	Arterial hypertension and dyslipidemia → C10	1.30	0.57	0.90
	Obstructive sleep apnea, arterial hypertension, and dyslipidemia → C10	1.30	0.43	0.90
	Arterial hypertension and dyslipidemia → C09	1.29	0.59	0.93
	*Dyslipidemia* → *C10*	*1.28*	*0.60*	*0.88*
	Obstructive sleep apnea and dyslipidemia → C10	1.27	0.46	0.88
	Obstructive sleep apnea, arterial hypertension, and dyslipidemia → C09	1.27	0.45	0.92
	Diabetes → C09	1.25	0.40	0.90
	*Arterial hypertension* → *C09*	*1.24*	*0.68*	*0.89*
	Dyslipidemia → C09	1.21	0.60	0.87
	Obstructive sleep apnea and arterial hypertension → C09	1.21	0.53	0.88
	Obstructive sleep apnea and dyslipidemia → C09	1.20	0.45	0.86

^a^A10: drugs used in diabetes.

^b^C09: agents acting on the renin-angiotensin system.

^c^C10: lipid-modifying agents.

^d^A02: drugs for acid-related disorders.

^e^N06: psychoanaleptics.

## Discussion

We hypothesized that disease-drug association rules in an underdiagnosed disease such as OSA can be a useful technique for inferring meaningful relations and filling information gaps in EHRs. We found three disease-drug association rules that, although not representing causal effects, were clinically confirmed by an expert on the topic.

Though this technique is not novel, we did not find studies performing this technique in OSA, but we did find it being used for cancer [6], drug alerts [5], diabetes [7], and heart failure [8]. All studies [5-8] used a medication list like ours, with some authors [7,8] stating that patients with an accurate and complete medication list may receive higher care. Also, they confirmed [5-8] that this technique is valid for identifying and filling gaps in the records, as well as benefiting the patient, as a mismatch within the records can indicate a medication error.

One of the limitations of this study is that we only used one researcher to manually extract nonstructural data (possible collection bias). Nevertheless, if we had only gathered information from the *International Classification of Diseases, Ninth Revision* codes, we would have missed relevant information.

To conclude, since ATC codes are used internationally and, to the best of our knowledge, there is no significant heterogeneity in their worldwide applicability in practice, this study demonstrates that drugs can be used to infer several active diseases and vice versa. Now, when a patient with suspected OSA has been prescribed a particular drug for a particular disease over a sustained time, we can confidently impute disease information with an error ranging from 5% (for the disease-drug association between agents acting on the renin-angiotensin system [C09] and arterial hypertension) to 13% (for lipid-modifying agents [C10] and dyslipidemia). Future work will measure the impact of using this imputation in Bayesian network model construction for OSA screening.

## Abbreviations

ATC: Anatomical Therapeutic Chemical
EHR: electronic health record
OSA: obstructive sleep apnea
